# A Dynamic Framework for Modelling Set-Shifting Performances

**DOI:** 10.3390/bs9070079

**Published:** 2019-07-18

**Authors:** Marco D’Alessandro, Luigi Lombardi

**Affiliations:** Department of Psychology and Cognitive Science, University of Trento, 38068 Rovereto, Italy

**Keywords:** Latent Markov Model, set-shifting, Wisconsin Card Sorting Test, Dynamical Systems, Psychological Assessment

## Abstract

Higher-order cognitive functions can be seen as a class of cognitive processes which are crucial in situations requiring a flexible adjustment of behaviour in response to changing demands of the environment. The cognitive assessment of these functions often relies on tasks which admit a dynamic, or longitudinal, component requiring participants to flexibly adapt their behaviour during the unfolding of the task. An intriguing feature of such experimental protocols is that they allow the performance of an individual to change as the task unfolds. In this work, we propose a Latent Markov Model approach to capture some dynamic aspects of observed response patterns of both healthy and substance dependent individuals in a set-shifting task. In particular, data from a Wisconsin Card Sorting Test were analysed in order to represent performance trends in terms of latent cognitive states dynamics. The results highlighted how a dynamic modelling approach can considerably improve the amount of information a researcher, or a clinician, can obtain from the analysis of a set-shifting task.

## 1. Introduction

In recent years there has been an increasing interest in modelling behavioural data from experimental tasks aimed at investigating higher-level cognitive functions [1,2,3,4,5,6]. Generally, higher-order cognitive functions can be seen as a class of cognitive processes which are crucial in situations requiring a flexible adjustment of behaviour in order to correspond to changing demands of the environment [7]. They also allow previous experiences and feedback related information to be integrated in order to maximize optimal choices [8]. Deficits at this level of cognitive functioning can be observed in rather heterogeneous clinical populations [9,10,11], each characterized, ideally, by a different pattern of impaired psychological sub-processes (see for example [3]). The cognitive assessment of these functions often relies on tasks which admit a dynamic, or longitudinal, component requiring an individual to modify h(er/is) behaviour during the unfolding of the task. For instance, consider a general set-shifting framework in which participants learn to pay attention and respond to relevant stimuli features, while ignoring irrelevant ones, as a function of experimental feedback. Here, negative feedback should allow participants to conceive a feature as irrelevant, modifying their responses accordingly. In this context, observed response patterns could consist of the occurrences of casual errors, feedback-related errors, and perseverations on shifting tasks, to name a few (e.g., [12]). The basic idea is that these response patterns reflect the presence (or the absence) of a cognitive impairment, either at a functional or neural level [13].

In this paper we propose a latent variable approach to model cognitive performances on a typical set-shifting task from a group level perspective. The approach is applied to data from the Wisconsin Card Sorting Test (WCST; [12,14]), which provides a renowned tool to measure set-shifting, deficient inhibitory processes on the basis of environmental feedback in cognitive settings [15]. In general, the test consists of a target and a set of stimulus cards with geometric figures that vary according to three perceptual features. The task requires participants to find the correct classification principle by trials and errors using the examiner’s feedback. An intriguing, and underestimated, aspect of such experimental protocol regards how individual performances change as the task unfolds, plausibly due to “learning to learn” capacity [16] or shifting cognitive strategies [14]. According to our view, a formal analysis of this *performance trend* (see, for example, [16]) can reasonably provide a novel interesting metric for the cognitive assessment of this sort of test outcomes. Therefore, several dynamic models regarding decision-making [17], learning [18], risky behaviour [19] and categorization [20], have proven to be able in uncovering characteristics of cognitive functioning which could not be detected with a standard (static) analytic approach based on collecting summary measures of individuals’ responses.

In order to take dynamic properties into account in our context, we adopted a Latent Markov Model (LMM; [21,22]) perspective to assess the dynamics of a latent states process underlying the observed behaviour. The basic assumption is that participants may evolve in their latent characteristic/states during the unfolding of the task. Thus, rather than simply analysing how the observed responses configuration evolves during the unfolding of the task, our target becomes to model the entire evolution of the latent states underlying these responses. The idea that observed behaviour is the final result arising from two or more latent data-generating states is clearly not new (e.g., [23,24]). Here, the basic intuition consists in the fact that human cognition can be influenced not only by external task demands, which are usually known and observable, but also by unknown latent mental processes and brain states that dynamically change with time [25]. In our context, observing a changing trend in participants’ responses (e.g., increase in the occurrence of perseverative errors at a given phase of the task) should reflect the fact that a change in the latent process has occurred. The Latent Markov Model nicely captures this intuition by considering the observed responses as a *measure* of the underlying latent states, and by directly providing a unified account for the dynamic of the latent states process. The reader is referred to [6,26] for different model-based approaches to model cognitive phenomena related to that analysed in the present work.

The manuscript is organized as follows. The next section provides the basic features of the LMM framework. In the third section, a model application is presented on a real data set collected using the WCST in the context of substance addiction. The last two sections present discussions of results and some conclusions.

## 2. Materials and Methods

### 2.1. The Formal Framework

Latent Markov models (LMM) were primarily developed for the analysis of longitudinal data, and thought to deal with categorical response variables [21]. Generally, a LMM can be seen as a generalization of the latent class model [27] allowing each subject to move between latent classes during time. To do so, these models make use of time-specific latent variables which are assumed to be discrete. Below, we briefly outline the main properties of this modelling approach.

**Figure 1:**
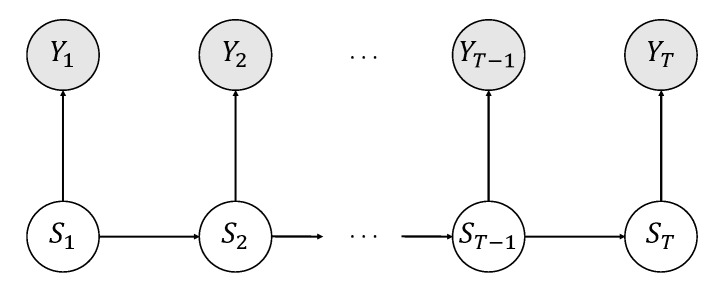
Conditional (in)dependencies structure of a basic LMM. Shaded nodes represent observed variables. White nodes represent unobserved (latent) variables.

To begin with, consider the directed graph depicted in Figure 1 illustrating the logic behind a basic LMM which evolves across *T* discrete time steps (e.g., [21,22]).

In a LMM, it is assumed that a sequence of observed response variables, $Y_{1},Y_{2},\ldots ,Y_{T}$, are conditionally independent given a corresponding pairwise sequence of latent variables, $S_{1},S_{2},\ldots ,S_{T}$, called *states*. More formally:

(1) $$\begin{array}{ccc} P(Y_{1},Y_{2},\ldots ,Y_{T}|S_{1},S_{2},\ldots ,S_{T}) & = & P\left(Y_{1}|S_{1}\right)P\left(Y_{2}|S_{2}\right)\cdots P\left(Y_{T}|S_{T}\right). \end{array}$$

The lack of directional connections (directed arrows) between observed variable nodes reflects the idea that only the latent states dynamics are responsible for the response pattern observed across the entire task. In other words, the evolution of the observed responses in time can be (phenomenologically) considered as the result of transition dynamics between latent states. In particular, the latent process follows a first-order Markov chain in that the latent variable $S_{t}$ at step *t* only depends on the outcome of the former step, $S_{t-1}$, (with t=2,…,T):

(2) $$\begin{array}{ccc} P(S_{1},S_{2},\ldots ,S_{T}) & = & P\left(S_{1}\right)\prod_{t=2}^{T}P\left(S_{t}|S_{t-1}\right). \end{array}$$

There are at least three main reasons why we consider this modelling approach promising in representing cognitive behaviours observed in set-shifting tasks: (1) a LMM provides a formal representation of the latent states that can be put in relation with a certain observed behaviour outcome [28] (2) it is a method to shape and analyse the unfolding of behaviour in time and its relation with the evolving dynamic of some aspects of cognition (3) it has a clear probabilistic framework to examine how different intervening factors (e.g., observed covariates) affect the evolving response patterns. Understanding the advantages of such general framework to model complex cognitive phenomena may be of great interest as discrete latent states can be nicely associated with some brain, cognitive, or abstract states that we assume might influence a given observed behaviour.

### 2.2. Model Application

In this section, we present the proposed modelling approach to analyse participants’ performances in the WCST and show how the LMM framework can account for differences between dynamic patterns in distinct groups. To this purpose, we apply the model to the analysis of an already published dataset (see [6,9]) that, in our context, represents an ideal case of set-shifting task study.

### 2.3. Participants

In our study, we analysed performances of 38 substance dependent individuals (SDI) and 44 healthy individuals in the Wisconsin Card Sorting Test (*ibidem*). Control participants had no history of mental retardation, substance abuse, or any systemic central nervous system disease. Regarding the SDI, the Structured Clinical Interview for DSM-IV [29] was used to determine a diagnosis of substance dependence. All participants in the study were adults (>18 years old) and gave their informed consent for inclusion which was approved by the appropriate human subject committee at the University of Iowa (see [9] for details).

### 2.4. Task Procedure

**Figure 2:**
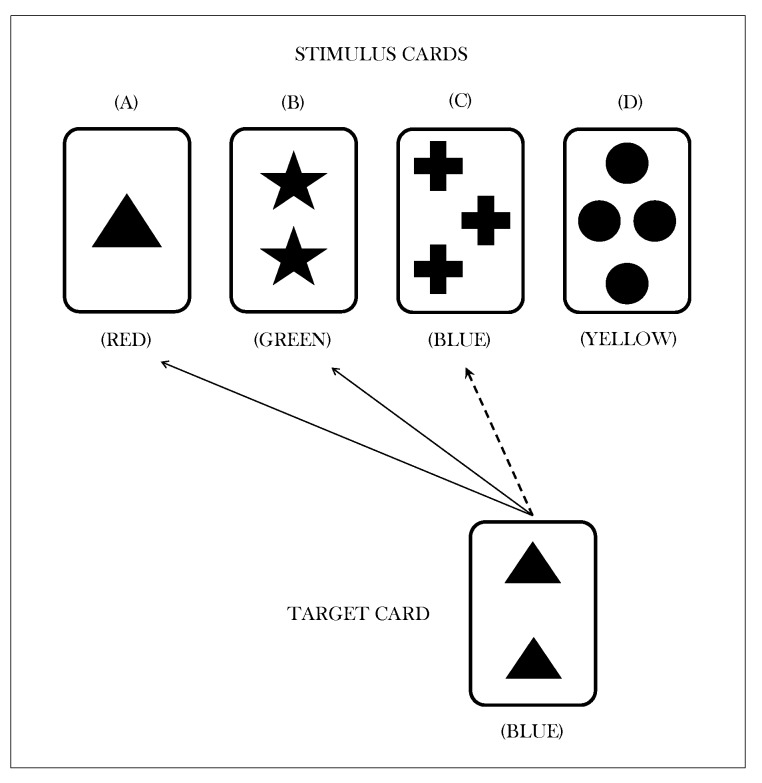
Example of a typical trial in the Wisconsin card sorting test. Arrows represent possible choices. In this example, the current sorting principle is *color*. Solid arrows, which sort the target card with stimulus cards (A) and (B), represent wrong matches. The dotted arrow, which sortes the target card with stimulus card (C), represents a right match.

In the common version of the WCST, participants are presented a target card and a set of four stimulus cards. All the cards consist of geometric figures that vary in terms of three features, namely, color (red, green, blue, yellow), shape (triangle, star, cross, circle) and number of objects (1, 2, 3 and 4). Figure 2 illustrates an example of a typical WCST trial. For each trial, a participant is asked to sort the target card with one of the four stimulus cards according to one of the three sorting rules. Each participant’s response is followed by a feedback (either positive or negative) telling the individual if the sorting is right or wrong. After a fixed number of consecutive correct responses, the experimenter changes the sorting rule without any warning to the participant. For each trial, the observed response can be classified as either a correct response, a perseverative error, or a non-perseverative error (see [12] for details). The error-related information is particularly meaningful as it seems to predict executive function deficits and frontal lobe dysfunctions (e.g., [30]). Moreover, the accounting of sub-types of error may help in discriminating cognitive processes that disrupt set-shifting performances in clinical population (e.g., [31]).

### 2.5. Data Modelling

In order to model the performance trends, we relied on the following data transformation procedure. First, we codified the observed sequence of participants’ responses according to a neuropsychological criterion proposed by Flashman, Horner, & Freides [32]. In particular, we focused on three categories of responses: *correct responses* (C), *non-persevertive errors* (E), and *perseverative errors* (PE). As a further step, for each participant, we considered the entire response pattern as partitioned into a limited number of blocks, also defined as *windows of trials*. Our main purpose was to model the dynamics of participants’ response patterns across these trial windows, rather than single trials. To this aim, in our application we considered for each participant five distinct windows (see Supplementary Material for details), which are thought to partition the entire task into (virtual) phases. More precisely, let $Z^{\left(j\right)}$ be the vector of responses for the individual *j*, such that, $Z^{\left(j\right)}\in \left\{C,E,\mathrm{PE}\right\}$. The response vector is partitioned as follows:

$$Z^{\left(j\right)}=\left(\left(z_{1}^{\left(j\right)},\ldots ,z_{n_{j}}^{\left(j\right)}\right),\left(z_{n_{j}+1}^{\left(j\right)},\ldots ,z_{2n_{j}}^{\left(j\right)}\right),\ldots ,\left(z_{4n_{j}+1}^{\left(j\right)},\ldots ,z_{5n_{j}}^{\left(j\right)}\right)\right)$$

where the element $z_{t}^{\left(j\right)}$ reflects the individuals’ codified response at trial *t*. The subscript $n_{j}$ indicates the length of the task phase for individual *j*, and is calculated in order to obtain equally-sized trial windows. It is important to notice that participants can vary in the number of observations within windows. The fact that participants are not homogeneous in the number of trials which constitute each phase is not a matter of concern for our modelling aim, since the task phases are considered to reflect the percentage of progress in the task. At this point, the resulting data structure was organized according to a longitudinal design where a specific block, $Y_{t}$, consisted of all the observed responses aggregated across all participants for a specific task phase. As an example, consider the data vector for the time occasion t=1, that is, for the first block of the longitudinal design. It consists of the aggregated responses of all participant’s first task phases, and can be formally represented as:

$$Y_{1}=\left(\left(z_{1}^{\left(1\right)},\ldots ,z_{n_{1}}^{\left(1\right)}\right),\left(z_{1}^{\left(2\right)},\ldots ,z_{n_{2}}^{\left(2\right)}\right),\ldots ,\left(z_{1}^{\left(J\right)},\ldots ,z_{n_{J}}^{\left(J\right)}\right)\right).$$

About the latent process characterization, we adopted a model selection criterion to choose the number of latent states *S*. Since our dependent variable is a categorical response variable with three levels, the possible choices reduce to a two-state model and a three-state model. In order to select the best model we relied on both BIC (Bayesian Information Criterion; [33]) and AIC (Akaike information criterion; [34]) criteria. Note that, for both criteria smaller values indicate a better model performance. Both two-state and three-state models are preferable to a baseline 1-state model which does not account for latent process dynamics (Table 1).

However, since results are very similar for the two candidate models, we adopted a further qualitative model selection criterion. In particular, we compared the estimates for the two models to determine which one provided the most useful and realistic substantive description of the data. We concluded that the three-state model accounted for a more sensible and complete description of set-shifting performances (see [35] for a similar approach). The reader is referred to the Supplementary Material for a more detailed comparison of models’ estimates. Thus, we required the model to be based on three distinct latent components, which were expected to have a direct psychological interpretation (see the results section). Moreover, in order to account for group differences in the latent process we also used a binary time-fixed covariate *X*, codifying the membership of each participant to either Control group (X=0) or Substance Dependent group (X=1). In such a way, we could control for eventual differences between the two sub-populations. Therefore, eventual differences in set-shifting performance trends between the two groups were completely captured by differences in the latent states dynamics (see Appendix A for details).

In what follows, we describe the model parameters and the main probabilistic relations in the system:

*(i)*  the *conditional response probabilities*
  $$\phi_{y|s}=P\left(Y_{t}=y|S_{t}=s\right),$$
  where y∈{C,E,PE} and s=1,2,3. This parameters set characterizes the measurement model which concerns the conditional distribution of the possible responses given the latent process. It is assumed that the measurement model is conditionally independent of the covariate. Here we are not interested in explaining heterogeneity in the response model between the two groups, since in our view only dynamics in the latent process are responsible for differences in performance trend between groups;
*(ii)*  the *initial probabilities*
  $$\begin{array}{ccc} \pi_{s|0} & = & P(S_{1}=s|X=0),\\ \pi_{s|1} & = & P(S_{1}=s|X=1), \end{array}$$
  where s=1,2,3. This parameter characterizes a distribution for the initial state across the (latent) states. In particular, $\pi_{s|0}$ and $\pi_{s|1}$ refer to the initial probabilities vectors of the states for the control group and for the substance dependent group, respectively;
*(iii)*  the *transition probabilities*
  $$\begin{array}{ccc} \pi_{s_{t}|s_{t-1}}^{\left(0\right)} & = & P(S_{t}=s_{t}|S_{t-1}=s_{t-1},X=0),\\ \pi_{s_{t}|s_{t-1}}^{\left(1\right)} & = & P(S_{t}=s_{t}|S_{t-1}=s_{t-1},X=1), \end{array}$$
  where t=2,…,5 and $s_{t},s_{t-1}=1,2,3$. This parameter characterizes the conditional probabilities of transitions between latent states across the task phases. In particular, $\pi_{s_{t}|s_{t-1}}^{\left(0\right)}$ and $\pi_{s_{t}|s_{t-1}}^{\left(1\right)}$ refer to the transition probabilities for the control group and the substance dependent group, respectively. Here we assume that a specific covariate entails the characterization of a sub-population with its own initial and transition probabilities of the latent process. In this way, accounting for differences in performance trend relies on explaining heterogeneity in the latent states process between the two groups;

According to this framework, the identification of the probabilistic relationships between latent states and observed responses, as well as those between latent states themselves, conveys all the information needed to characterize the observed response patterns dynamics.

## 3. Results

The proposed model was fitted using the *LMest* package [36] developed within the *R* framework [37]. *LMest* relies on an efficient log-likelihood maximization procedure (e.g., Expectation- Maximization Algorithm) for parameters estimation. Moreover, a model selection criterion was used to evaluate if the model with the group covariate *X* was preferable to the simpler model without the grouping variable. In particular, we adopted both the BIC (Bayesian Information Criterion; [33]) and AIC (Akaike information criterion; [34]) to measure the overall model performance. As expected, the model with the group covariate turned out to be the most appropriate model (see Table 2) thus confirming that the performance patterns were clearly different between the two groups.

### 3.1. Conditional Response Probabilities

The estimated conditional response probabilities ${\hat{\phi }}_{y|s}$ are presented in Table 3. These probabilities allowed us to characterize the latent states. The first state (s=1) showed the highest probability to respond correctly, indicating that participants minimized errors within a task phase. By contrast, the second state (s=2) showed an increased probability of the error component, in particular the probabilities that a non-perseverative error or a perseverative error occur were approximately the same. This indicated the adoption of a non-efficient strategy, although the probability to respond correctly was still relatively high. Finally, the third state (s=3) showed a different pattern in which the probability to produce a correct response resulted lower than the probability to produce an error. The errors pattern also entailed a higher perseverative component compared to the second state.

These probability distributions represent cognitive response strategies as a function of the latent component or state. In particular, in our context, State 1 may be easily understood as an *Optimal Strategy* whereas State 2 seems to characterize a type of *Sub-Optimal Strategy*. Finally, State 3 indicates a *Perseverative Non-Optimal Strategy*. Therefore, this latent states characterization may be adopted to describe the average ability to operate shifting cognitive strategies.

### 3.2. Initial Probabilities

Table 4 reports the model initial probability configurations. These initial probabilities indicated that the two groups performed the early phase of the test very differently. In particular, the control group showed a higher overall probability of starting the initial test phase in State 1. By contrast, the SDI group showed a higher probability to adopt a strategy admitting an error component at the initial phase of the task. This interesting result could reflect the finding that substance dependent individuals usually show an inefficient initial conceptualization of the task [12].

### 3.3. Transitions Probabilities

**Figure 3:**
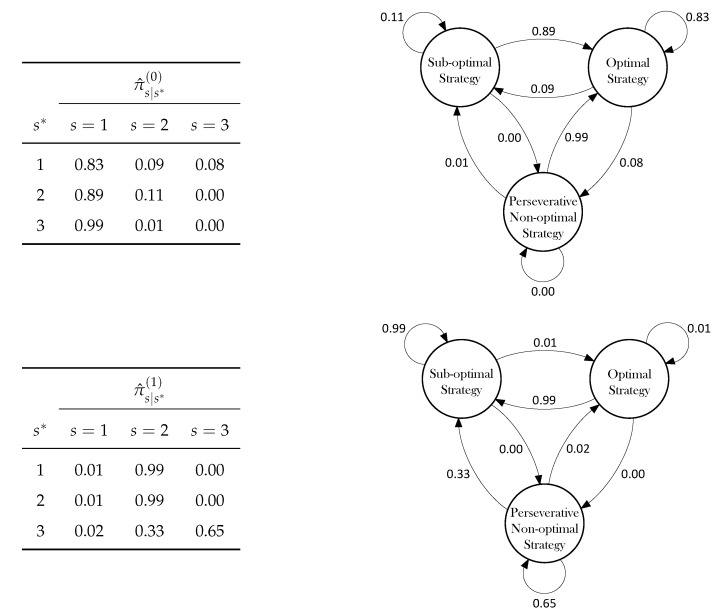
Transition probability matrices (**left**) and relative graphical model representations (**right**) for the control group (**top**) and the SDI group (**bottom**).

All the available information on the dynamics of the latent process can be conveyed by the transition probabilities matrices (see Figure 3). These matrices represent, at a given task phase *t*, the probability to transit from a current state *s* to a different state $s^{*}$ or to remain in the same state *s*.

The transition matrix for the control group showed a clear pattern. First, the diagonal values revealed that the probability to reiterate a certain strategy decreased as it became less optimal, up to a zero probability to reiterate transitions to State 3, which clearly represents the non-optimal response strategy. Moreover, the very low probability values in the second and third columns indicate that it was nearly impossible to adopt a response strategy affected by the error component, since the overall probabilities to transit to State 2 or State 3 approached zero. It is also worth noting that, the probabilities approaching 1 in the first column indicate a general tendency of the system to transit to State 1, suggesting that these participants tended to switch to the optimal strategy in case they were not in that status, and to maintain that strategy for the rest of the task. Clearly, this pattern reflected the tendency to quickly minimize both the perseverative and the non-perseverative components of the error, as the task unfolded across time.

The transition matrix for the SDI group showed rather different dynamics. Importantly, the system exhibited a general tendency to transit to State 2, the sub-optimal strategy. In particular, it is worth emphasizing that a probability approaching 1 on the main diagonal could be understood as the presence of an *absorbing state*. This means that once in State 2, the system tended to reiterate the same latent state and that SDI participants systematically reiterated the sub-optimal strategy and never transited to the optimal strategy during the task. Further, once in State 3, there was a relatively high probability that an individual remained stacked in that state, indicating the tendency to reiterate the non-optimal strategy and to show a perseverative component of the error. On one hand, this pattern could also reflect the presence of mental rigidity as for substance dependent individuals was nearly impossible to switch to the optimal strategy. On the other hand, the tendency to reiterate a sub-optimal strategy by keeping fixed the error component across the task could also be seen as a probabilistic account for the failure to maintain set phenomenon [38]. This is in accordance with some findings reporting this peculiar behaviour in substance dependent individuals [16].

### 3.4. Marginal Latent States Distributions

**Figure 4:**
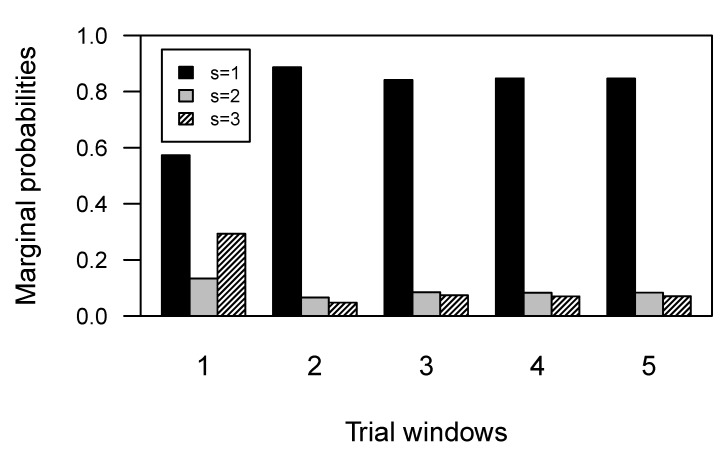
Marginal distribution of the latent states for each task phase, for the Control group.

**Figure 5:**
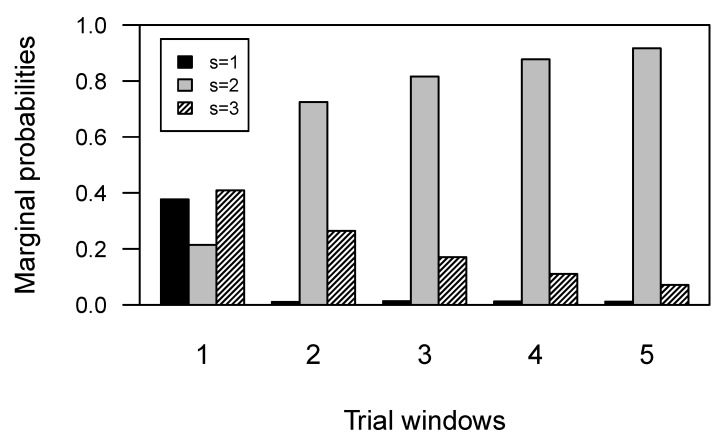
Marginal distribution of the latent states for each task phase, for the SDI group.

In order to better understand our model results, we analysed the marginal distribution of the latent states. For each task phase, we derived a probability distribution over the three states for each group. To do so, we relied on basic rules for markov chains. Let $\pi_{t}$ be the distribution of the latent states at a certain time step *t*, or task phase, and let the transition matrix $\pi_{s_{t}|s_{t-1}}$ be codified as *P*, for notational convenience. For each time step t+1,t+2,…,T=5 we want to compute the quantities $\pi_{t+1},\pi_{t+2},\ldots ,\pi_{T}$. The purpose is to move the distribution $\pi_{t}$ forward one unit of time, by starting from $\pi_{1}$, which is the initial probability vector. It can be shown that $\pi_{t+n}=\pi_{t+(n-1)}P$ [39]. Regarding the control group, Figure 4 shows that the optimal strategy was maintained for the entire duration of the test, and the probability to adopt a strategy admitting errors component decreased quickly. This indicates that participants in the control group tended to learn immediately how to minimize the error component. Figure 5 shows the marginal distributions of the states for the SDI group. The plot shows that the probability to adopt an optimal strategy decreased faster than the probability to adopt a non-optimal perseverative strategy. The sub-optimal strategy with both error components showed the higher probability to be maintained for the rest of the task, suggesting that substance dependent individuals never minimized the error component during the test.

## 4. Discussion of Results

Results clearly show that our model is able to capture differences in performance trend between Control and SDI groups in terms of differences in their latent process transition dynamics. The characterization of the conditional response probabilities allows to rephrase the latent states as cognitive strategies adopted in a given phase of the task. Results also show that a three-state model is a reasonable choice if we want to differentiate dynamics in strategy shifting between groups. In fact, it is unrealistic to think that individuals can rely only on two (latent) cognitive strategies to accomplish the task, as it would be in case of a two-state model. The three states could be clearly interpreted as error-related strategies with gradually increasing error components. However, one might argue that our state process characterization does not account for three distinct latent components, due to similarities in probability patterns of responses for some of the states (such as State 1 and 2). According to our view, an inspection of the marginal distributions of the latent states in Figure 4 and Figure 5 can clarify that our model actually accounts for three non-overlapping latent components, as reflected by the differential marginal states probabilities pathways for the two groups.

It is also worth emphasizing that these results can increase the amount of information a researcher can obtain from the assessment of set-shifting performances. Generally, the analysis of data from the WCST reduces to the computation of summary statistics of the scoring measures, which in turn may provide the input for standard statistical analysis, as well as for classification procedures based on cut-off thresholds [15]. Mean scoring measures across individuals provide a simple way to account for group-level differences in performances (Table 5).

In our case study the groups differ in the number of perseverative (t(80)=5.62, p<0.001) and non-perseverative (t(80)=6.48, p<0.001) errors. However, mean differences cannot account for hypothesis about the underlying causes. From our modelling perspective, differences in mean scoring measures can be explained by the heterogeneity in the latent process affecting the way in which individuals within each group respond at a given phase of the task. Thus, a fundamental additional information provided by our model consists in the data generating process.

## 5. General Discussion

The modelling approach proposed in this work was able to map the evolution of response patterns in a set-shifting task with the evolution of a latent states process underlying the observed behaviour. The model provided a parsimonious description of the dynamic processes underlying the data, since we were able to represent the performance trend by using a latent variable with just three categories, representing different cognitive strategies evolving in time. Moreover, the estimated parameters capturing these dynamic aspects could be readily put in relation with some psychological constructs of potential clinical relevance. However, a crucial issue is that related to the interpretation of these parameters. Although accounting for a data generating process could convey interesting and additional information for the analysis of behaviour outcomes, parameters interpretation is not trivial. Marginal latent states distributions offered a straightforward way to examine dynamic aspects of error-related behaviour. For instance, marginal distributions showed that control group (resp. SDI group) settles to State 1 (resp. State 2) across task phases, which is approximately the same information conveyed by the summary measures of the number of errors for each group. From this perspective, marginal distributions provided no additional information for the analysis of participants’ performances. Conversely, transition probabilities matrices provided a more exhaustive source of information at the cost of an increasing difficulty in results interpretation (e.g., differences in performance trends between groups must rely on row-wise, column-wise, main diagonal values comparison). Therefore, transition probabilities offer the advantage to rely on parameters estimates for simulation and forecasting purposes. In particular, the transition matrices can be seen as cognitive system profiles and one might be interested in generating data in order to test sensible hypothesis. For example, given the two estimated profiles, one for each group, a sensible question could be: Which system does reach the optimal strategy first, on average, given the assumption that both systems start the task at State 3, the perseverative non-optimal strategy? This kind of investigation could be hard, or even impossible, for standard analytical frameworks based on simple summary statistics of the scoring measures.

In conclusion, our LMM model provides, at least in this first preliminary work, an interesting tool to analyse data presenting a dynamic component. It also illustrates an efficient way to manage differences between groups by accounting for the heterogeneity in the latent process characteristics between them. However, further works are needed in order to solidly establish connections between parameters estimates and more subtle cognitive constructs.

## Figures and Tables

**Table 1:** Latent States selection.

Model	BIC	AIC
1-state	8792	8781
two-state	8561	8490
three-state	8608	8493

**Table 2:** Model Selection criteria.

Model	BIC	AIC
Basic	8858	8691
Covariate	8608	8493

**Table 3:** Estimated conditional probabilities of responses given the latent state.

	${\hat{\phi }}_{y|s}$		
y	s=1	s=2	s=3
C	0.93	0.80	0.44
E	0.02	0.10	0.38
PE	0.05	0.10	0.18

**Table 4:** Estimated initial probabilities for each group.

	s=1	s=2	s=3
${\hat{\pi }}_{s|0}$	0.57	0.14	0.29
${\hat{\pi }}_{s|1}$	0.38	0.21	0.41

**Table 5:** Mean scoring measures (*SE* in parenthesis).

	*C*	*E*	*PE*
Control	66.34 (0.64)	5.25 (0.29)	4.65 (0.42)
SDI	72.34 (2.17)	10.57 (0.96)	14.28 (1.52)

## Supplementary Materials

The following are available online at https://www.mdpi.com/2076-328X/9/7/79/s1.

## Abbreviations

The following abbreviations are used in this manuscript:

LMM	Latent Markov Model
WCST	Wisconsin Card Sorting Test

## Appendix A

In order to allow the covariate to condition the characterization of the latent process we adopt a logit parameterization as follows:

$$\log\frac{P(S_{1}=s|X_{t}=x)}{P(S_{1}=1|X_{t}=x)}=\log\frac{\pi_{s|x}}{\pi_{1|x}}=x^{⊤}\Theta$$

where s=2,…,k, for the initial probabilities, and

$$\log\frac{P(S_{t}=s|S_{t-1}=s^{*},X_{t}=x)}{P(S_{t}=s^{*}|S_{t-1}=s^{*},X_{t}=x)}=\log\frac{\pi_{s|s^{*},x}}{\pi_{s^{*}|s^{*},x}}=x^{⊤}\Gamma$$

where t=2,…,T=5, $s,s^{*}=1,2,3$ and $s\neq s^{*}$, for the transition probabilities. In both parameterization, $x^{⊤}$ is a proper design matrix, whilst Θ and Γ are regression coefficients vectors.

In this way the covariate entails the characterization of a sub-population with its own initial and transition probabilities of the latent process, whereas the conditional distribution of the response variable given the latent process does not depend on the specific sub-population.
