# Supplementary material for: A Dynamic Framework for Modelling Set-Shifting Performances
Source: Behav Sci (Basel). 2019 Jul 18;9(7):79. doi: 10.3390/bs9070079 (PMC6680592; doi:10.3390/bs9070079)
Supplement: Supplementary file 1 [file behavsci-09-00079-s001.pdf]

A dynamic framework for modeling set-shifting performances: Supplementary Material

Marco D'Alessandro and Luigi Lombardi

Department of Psychology and Cognitive Science

University of Trento

Send all correspondence to:

Marco D'Alessandro

Department of Psychology and Cognitive Science

Corso Bettini 31

I-38068 Rovereto (TN), Italy

Email: marco.dalessandro@unitn.it

Selection criteria for the number of Trial Windows

In a typical Wisconsin Card Sorting Test it is often the case in which a great heterogeneity in the number of trials needed to accomplish the task is observed between individuals. In our group-level modelling proposal we adopted a data transformation procedure to obtain a longitudinal structure in which each block had to contain the same number of data points. In this way the aggregated vector of responses for the i-th trial window, across individuals, is mapped to the i-th longitudinal block Y_i. The more natural way to capture changes in set-shifting performances could consist in organizing the longitudinal data structure by partitioning the vector $Z^{(j)}$ in order to take a specific number of trials after a change of the sorting rule occurs. However, individuals in our study differed in the number of trials achieved to complete a category, that is, before a change of the sorting rule occurs. A trial windows clustering based on selecting a specific number of trials after a change of the sorting rule does not ensure the regularity of the longitudinal structure, due to the individual variability in completing a category.

In our model, the windows were equally sized, and the choice of the number of windows, T, directly affected the number of trials within them. For this reason, some trials had to be excluded when the total number of trials achieved by a given participant was not a multiple of T. There are two main reasons why we fixed T=5:

1. First, we selected the value of T which ensured the least data points loss for the aggregated dataset of healthy and substance dependent individuals (data points removed: 1.9% for T=5, 2.9% for T=6, 2.5% for T=7).

2. The computational machinery of LMM needs longitudinal structures with a great number of observations within a specific longitudinal block (Bartolucci et al., 2012). The model with T=5 maximizes the number of data points within the longitudinal blocks, by ensuring a more reliable parameters estimates.

Conditional Response Probabilities comparison

The following tables show the conditional response probabilities estimates for three models with different number of states (1-state, left; 2-state, center; 3-state, right).

	$\hat{\phi}_{y\|s}$
y	$s=1$
C	0.80
E	0.11
PE	0.09

	$\hat{\phi}_{y\|s}$	
y	$s=1$	$s=2$
C	0.92	0.67
E	0.02	0.20
PE	0.06	0.13

	$\hat{\phi}_{y\|s}$		
y	$s=1$	$s=2$	$s=3$
C	0.93	0.80	0.44
E	0.02	0.10	0.38
PE	0.05	0.10	0.18

The 1-state model can be considered as a baseline model which accounts for the absence of dynamics in the performance trend. The 2-state and the 3-state models are the candidate models in the main work. Our qualitative model selection criteria relies on comparing their conditional probabilities matrices. As can be noticed, the State 2 in the 2-state model reflects the error-related cognitive strategy. However, in our view, the error-related strategy can be decomposed in order to obtain two types of non-optimal strategies accounting for different degree of non-perseverative and perseverative components of the error. A discussion on this point can be found in the "Discussion of Results" section in the main manuscript.

References

Bartolucci, F., Farcomeni, A., Pennoni, F. (2012). Latent Markov Models for longitudinal data. Chapman and Hall/CRC press.
